# Action Potential Clamp and Pharmacology of the Variant 1 Short QT Syndrome T618I hERG K^+^ Channel

**DOI:** 10.1371/journal.pone.0052451

**Published:** 2012-12-26

**Authors:** Aziza El Harchi, Dario Melgari, Yi Hong Zhang, Henggui Zhang, Jules C. Hancox

**Affiliations:** 1 School of Physiology and Pharmacology and Cardiovascular Research Laboratories, University of Bristol, Bristol, United Kingdom; 2 Biological Physics Group, School of Physics and Astronomy, The University of Manchester, Manchester, United Kingdom; Sackler Medical School, Tel Aviv University, Israel

## Abstract

**Background:**

The familial Short QT Syndrome (SQTS) is associated with an increased risk of cardiac arrhythmia and sudden death. Gain-of-function mutations in the hERG K^+^ channel protein have been linked to variant 1 of the SQTS. A hERG channel pore (T618I) mutation has recently been identified in families with heritable SQTS. This study aimed to determine effects of the T618I-hERG mutation on (i) hERG current (I_hERG_) elicited by ventricular action potentials; (ii) the sensitivity of I_hERG_ to inhibition by four clinically used antiarrhythmic drugs.

**Methods:**

Electrophysiological recordings of I_hERG_ were made at 37°C from HEK 293 cells expressing wild-type (WT) or T618I hERG. Whole-cell patch clamp recording was performed using both conventional voltage clamp and ventricular action potential (AP) clamp methods.

**Results:**

Under conventional voltage-clamp, WT I_hERG_ peaked at 0-+10 mV, whilst for T618I I_hERG_ maximal current was right-ward shifted to ∼ +40 mV. Voltage-dependent activation and inactivation of T618I I_hERG_ were positively shifted (respectively by +15 and ∼ +25 mV) compared to WT I_hERG_. The I_hERG_ ‘window’ was increased for T618I compared to WT hERG. Under ventricular AP clamp, maximal repolarising WT I_hERG_ occurred at ∼ -30 mV, whilst for T618I hERG peak I_hERG_ occurred earlier during AP repolarisation, at ∼ +5 mV. Under conventional voltage clamp, half-maximal inhibitory concentrations (IC_50_) for inhibition of I_hERG_ tails by quinidine, disopyramide, D-sotalol and flecainide for T618I hERG ranged between 1.4 and 3.2 fold that for WT hERG. Under action potential voltage clamp, T618I IC_50_s ranged from 1.2 to 2.0 fold the corresponding IC_50_ values for WT hERG.

**Conclusions:**

The T618I mutation produces a more modest effect on repolarising I_hERG_ than reported previously for the N588K-hERG variant 1 SQTS mutation. All drugs studied here appear substantially to retain their ability to inhibit I_hERG_ in the setting of the SQTS-linked T618I mutation.

## Introduction

The rapid delayed rectifier K^+^ channel current (I_Kr_) is an important determinant of ventricular AP repolarisation and, consequently, of the duration of the QT interval on the electrocardiogram [1], [2]. Channels mediating I_Kr_ are formed by proteins encoded by *hERG* (*human Ether-à-go-go Related Gene;* alternative nomenclature *KCNH2* [3], [4]). Native I_Kr_ and hERG channels exhibit sensitivity to pharmacological blockade by diverse drugs, including both Class Ia and Class III antiarrhythmic agents; excessive pharmacological inhibition of I_Kr_/hERG leads to acquired long QT syndrome (LQTS [5]–[8]). Loss-of-function *KCNH2* mutations are responsible for the LQT2 form of heritable long QT syndrome [9], [10], whilst gain-of-function mutations are responsible for the SQT1 form of heritable Short QT syndrome (SQTS [11], [12]).

The *KCNH2* mutations first identified in SQTS patients led to a common asparagine to lysine (N→K) substitution within the external S5-Pore linker region of the hERG channel protein [13], [14]. hERG current (I_hERG_) carried by N588K-hERG mutant channels failed to rectify normally, due to a substantial (+60 to +90 mV) rightward shift in voltage-dependent inactivation [13], [15], [16]. The use *in vitro* of the action potential (AP) voltage clamp technique showed that the impaired inactivation of N588K hERG channels altered significantly the profile of I_hERG_ during the plateau and repolarisation phases of ventricular APs, leading to increased I_hERG_ occurring much earlier during the ventricular AP waveform [13], [15], [16]. Additionally, SQT1 patients with the N588K mutation were found to be refractory to treatment with Class III antiarrhythmic drugs (sotalol, ibutilide), but did respond to the Class Ia agents quinidine and disopyramide [13], [17]–[19]. This differential influence of the N588K mutation on clinical effectiveness of Class Ia and III drugs correlates with changes in I_hERG_ blocking potency seen *in vitro* [13], [18], [20] and is explicable on the basis of the comparatively greater dependence of Class III than Class Ia drugs on I_hERG_ inactivation in order to bind to the channel [21]. A second gain-of-function hERG mutation, identified in the S5 domain of zebrafish ERG (zERG; L499P; hERG homologue L532P) in *reggae* mutant zebrafish with accelerated cardiac repolarisation [22], has been found to produce marked kinetic alterations including to voltage and time-dependent inactivation [22], [23]. The L532P hERG homologue also exhibits altered sensitivity to Class III drug block [23].

Recently, a novel SQT1 mutant has been identified in a Chinese family with a history of nocturnal sudden death [24]. Four of eleven family members evaluated exhibited shortened rate-corrected QT intervals (with a mean QT_c_ interval of 316 ms) [24]. Genotyping of the proband identified a base transition (C1853T) that led to a threonine to isoleucine substitution at position 618 (located in the hERG channel pore helix) of hERG; this was absent in 200 ethnically matched controls [24]. *In vitro* biophysical analysis identified significant alterations to I_hERG_ kinetics, including a ∼+50 mV shift in voltage dependent inactivation [24]. Pharmacological experiments with single high concentrations of quinidine or sotalol (producing 70% or greater inhibition of wild-type (WT) I_hERG_) were suggestive of retained I_hERG_ block of T618I hERG during applied voltage commands [24]. At present, however, concentration-response data for pharmacological inhibition of T618I hERG appear to be lacking for any drug. Moreover, the effect of the T618I mutation on the profile of I_hERG_ during dynamic physiological waveforms (ventricular APs) has not yet been reported. The present study was conducted to address both of these issues, through experiments on recombinant WT and T618I channel I_hERG_ conducted at human physiological temperature.

## Materials and Methods

### Wild-type and T618I hERG

Human Embryonic Kidney (HEK-293) cells stably expressing WT hERG were donated by Prof Craig January [25]. HEK 293 cells used for transient transfection were obtained from ECCAC (catalog number 85120602). The T618I mutant was constructed using QuikChange® (Stratagene) mutagenesis. The following forward primer sequence was used: 5′CGG CGC TCT ACT TCA TCT TCA GCA GCC TCAC3′. DNA was sequenced for the full length of the hERG insert to ensure that only the correct mutation had been made (Eurofins MWG Operon).

### Maintenance of Cells and Cell Transfection

Experiments employed HEK-293 cells stably or transiently expressing WT or T618I hERG constructs. Cells were passaged and maintained as described previously [23], [26]. For transient transfection experiments, 24 hours after plating cells out they were transiently transfected with 0.3 µg of T618I hERG construct using Lipofectamine™ LTX (Invitrogen) according to the manufacturer’s instructions. Expression plasmid encoding CD8 was also added as a transfection marker [26]. Cells were plated onto small sterilised collagen-coated glass coverslips 6 hours after transfection and recordings were made after at least 24 hours incubation at 37°C. Successfully transfected cells were identified using Dynabeads® (Invitrogen).

### Electrophysiology

Once in the recording chamber, cells were superfused at 37°C with an external solution containing (in mM): 140 NaCl, 4 KCl, 2.5 CaCl_2_, 1 MgCl_2_, 10 Glucose and 5 HEPES (titrated to pH 7.45 with NaOH). Patch-pipettes (Corning 7052 glass, AM Systems) were pulled and heat-polished (Narishige MF83) to 2.5–4 MΩ; pipette dialysate contained (in mM): 130 KCl, 1 MgCl_2_, 5 EGTA, 5 MgATP, 10 HEPES (titrated to pH 7.2 using KOH) [26]; [27]. hERG current (I_hERG_) recordings were made using an Axopatch 200, 200A or 200B amplifier (Axon Instruments, now Molecular Devices) and a CV201, CV202A or CV203BU head-stage. Between 70–80% of pipette series resistance was compensated. Voltage-clamp commands were generated and data recorded using ‘WinWCP’ (John Dempster, Strathclyde University) or pClamp 9.0 and 10.0 (Molecular Devices). The ventricular action potential (AP) command used for AP clamp experiments was identical to that used in other recent studies from our laboratory [23], [27].

### Drugs

Disopyramide-phosphate powder (Sigma-Aldrich) was dissolved in Milli-Q water to produce an initial stock solution of 400 mM which was diluted further to produce stock solutions ranging down to 1 mM. Quinidine gluconate salt (Sigma-Aldrich) was dissolved in MilliQ water to produce an initial stock solution of 100 mM, which was diluted further to produce stock solutions ranging down to 30 µM. Flecainide acetate salt (Sigma-Aldrich) was dissolved in MilliQ water to produce an initial stock solution of 10 mM, which was diluted further to produce stock solutions ranging down to 1 mM. D-sotalol (Sequoia) was dissolved in DMSO to produce an initial stock solution of 100 mM, with further dilution of stocks to solutions ranging down to 10 µM. Disopyramide and quinidine containing stock solutions were diluted at least 1∶1000-fold with Tyrode’s solution to achieve the final concentrations stated in the Results text. For D-sotalol, dilutions of 1∶1000 fold were achievable for all final concentrations except 500 µM, for which a dilution of only 5∶1000 fold was possible. During recordings all external solutions were applied using a home-built, warmed and rapid solution exchange device [28].

### Data Analysis

Concentration-response data were fitted by a standard Hill equation in order to obtain half-maximal inhibitory concentration (IC_50_) and Hill-coefficient (n_H_) values (±95% confidence intervals (C.I.)). Mean data are otherwise presented as mean ± SEM. The voltage dependence of I_hERG_ activation was determined by fitting the values of I_hERG_ tail currents (normalised to peak I_hERG_ tail value and plotted against voltage) with a Boltzmann equation of the form:
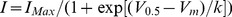
(1)where I is the I_hERG_ tail amplitude following test potential V_m_, I_Max_ is the maximal I_hERG_ tail observed during the protocol, V_0.5_ is the potential at which I_hERG_ was half-maximally activated, and *k* is the slope factor for the relationship.

The voltage dependence of I_hERG_ inactivation (assessed by studying availability) was determined by fitting normalised peak I_hERG_ currents elicited by the third step of a three-step protocol (Figure 3A) by the equation:
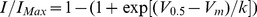
(2)where *I = *transient current elicited by the third step of the protocol, following a brief (2 ms) conditioning step (V_m_) to relieve inactivation induced by the first step; *I_Max_* is the maximal transient current observed during the protocol and *V_0.5_* and *k* denote, respectively, half-maximal inactivation voltage and slope factor for the fit to the plotted relation.

Statistical analysis (SigmaPlot 12) utilised, as appropriate, an unpaired t-test, a Welch’s t-test not assuming equal variances, or a two way repeated measures ANOVA test. *P* values of less than 0.05 were taken as statistically significant.

## Results

### Effects of the T618I hERG Mutation on the Voltage-dependence of I_hERG_ and its Activation

**Figure 1:**
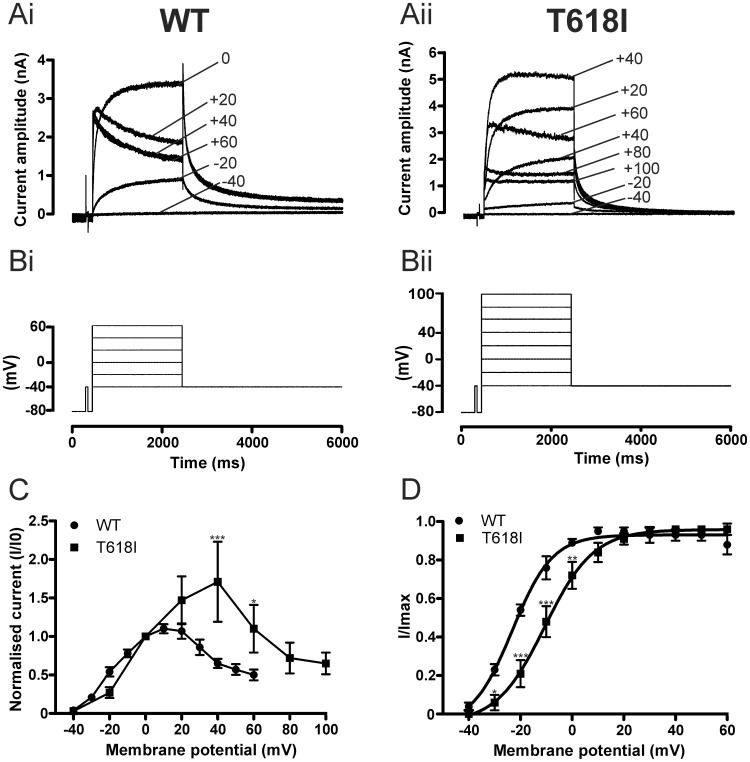
Basic characteristics of T618I I_hERG_. (**A,B**) Upper traces (**A**) show representative current records for WT I_hERG_ (Ai) and T618I I_hERG_ (Aii) elicited by voltage protocols shown in **B** (Bi – WT and Bii - T618I; note that for WT I_hERG_ measurements, successive voltage steps increased by 10 mV increments up to +60 mV, whilst for T618I I_hERG_ successive steps increased by 20 mV up to +100 mV). Note that for clarity of display only selected traces elicited by the protocol are shown. Numbers in Ai and Aii indicate command voltage at which currents recorded. Note different current scales in Ai and Aii. (**C**) Current voltage (I–V) relations for WT (n = 6) and T618I (n = 5) I_hERG_ elicited by the voltage protocol shown in panel B. Data are shown for end pulse I–V relations, in which currents for each cell studied were normalized to the current value at 0 mV. (**D**) I–V relations for WT (n = 6) and T618I (n = 7) tail current recorded at −40 mV following repolarisation from the test potentials plotted on the membrane potential axis (for these measurements I_hERG_ tails were measured following depolarising steps to potentials between −40 and +60 mV (in 10 mV increments)). Tail currents were measured relative to the instantaneous current elicited by the brief (50 ms) step from −80 to −40 mV prior to the applied voltage commands. Data were fitted by equation 1, to give the V_0.5_ and *k* values in the Results text. Asterisks in C and D denote statistical significance: *p<0.05; **p<0.01 ***p<0.001.

Figures 1Ai and Aii show representative current traces for WT and T618I I_hERG_ elicited by the voltage protocol shown in the lower panels (Figures 1Bi and Bii). WT I_hERG_ increased progressively with the magnitude of the applied voltage commands up to ∼0/+10 mV, positive to which the current during the applied command declined in amplitude. Prominent I_hERG_ tails were observed on repolarisation to −40 mV after each voltage command, with tail current amplitude exceeding that of the preceding current during the depolarising step, particularly for positive command voltages. These features are typical of WT I_hERG_ [3], [4], [25], [29]. The traces shown in Figure 1Aii indicate that at potentials negative to 0 mV, T618I I_hERG_ resembled WT I_hERG_. However, at test potentials between ∼ 0 and +40 mV (over which WT I_hERG_ elicited by depolarising commands became reduced in amplitude), T618I I_hERG_ continued to increase in magnitude. T618I I_hERG_ began to decline in amplitude at test potentials positive to +40 mV. Notably, with positive test commands, T618I I_hERG_ did not exhibit tail currents (I_tails_) that exceeded pulse current in amplitude (*cf* [24]). Figure 1C shows mean end-pulse current voltage (I–V) relations for WT and T618I I_hERG_, demonstrating maximal current for WT I_hERG_ at ∼+10 mV and an area of negative slope in the I–V relation at more positive potentials. For T618I hERG, rectification of the I–V relation was positively voltage-shifted, with the area of negative slope in the I–V relation occurring positive to +40 mV. Figure 1D shows mean normalised I–V relations for WT and T618I hERG I_tails_, fitted with equation 1 (Methods). For WT I_hERG_ the mean activation V_0.5_ was −23.1±1.5 mV (n = 6), whilst for T618I I_hERG_ this was −8.0±3.4 mV (n = 7; p<0.01 versus WT). The corresponding *k* values were 7.8±1.5 and 8.5±0.9 mV respectively (p>0.05).

### Effects of the T618I Mutation on I_hERG_ Activation and Deactivation Time-course

**Figure 2:**
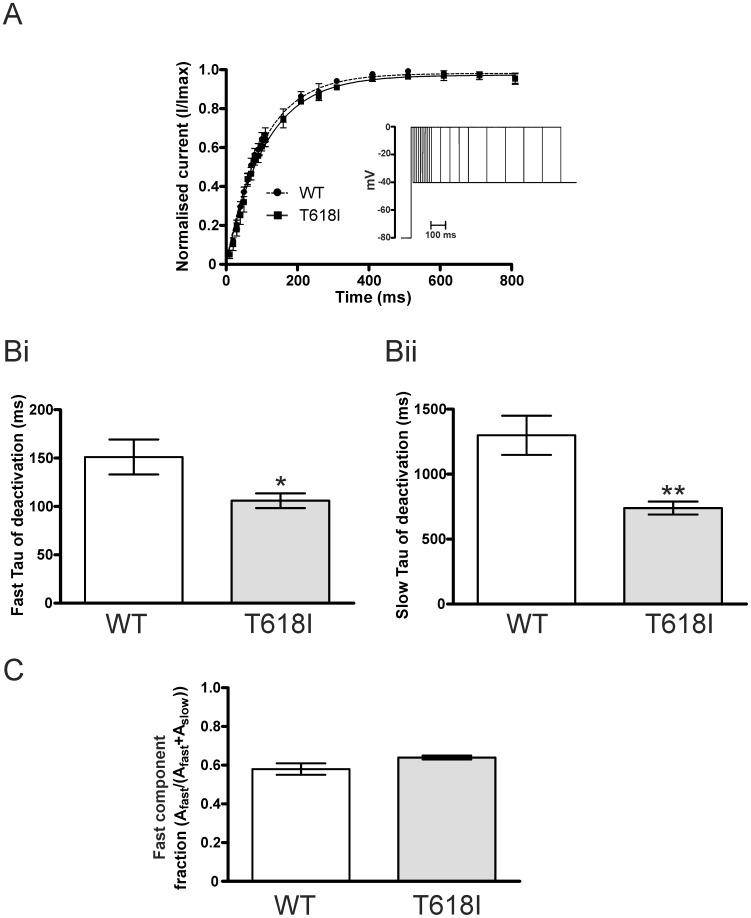
WT and T618I I_hERG_ time-course of activation and deactivation. (**A**) Plots of time-course of I_hERG_ activation obtained using an “envelope-of-tails” protocol– see inset and ‘Results’ text. For each cell, the peak current amplitudes at each time-point were normalized to the maximal current observed during the protocol. (**Bi, Bii**) Bar charts comparing τ_f_ (Bi) and τ_s_ (Bii) values for deactivation of WT (n = 11) and T618I (n = 11) hERG tail currents on repolarisation to -40 mV following a 2 s depolarisation from −80 mV to +20 mV. Currents were fitted with a standard bi-exponential equation (**C**) Bar-chart showing the proportion of fast deactivation on repolarisation to −40 mV for WT and T618I I_hERG_ (n = 11 cells for each condition). Asterisks in Bi and Bii denote statistical significance: *p<0.05; **p<0.01.

In order to investigate effects of the T618I mutation on the time-course of I_hERG_ activation, we used an “envelope of tails protocol” in which I_tails_ were measured at −40 mV following activating commands of different durations from −80 to 0 mV (see inset to Fig. 2A). I_tails_ elicited by commands of different duration were normalised to the maximum current during the protocol and plotted as a function of command pulse duration, as shown in Figure 2A (*cf* [23], [30]). Mono-exponential fits to the data yielded a τ_activation_ of 104.1±8.3 ms for WT I_hERG_ and of 112.0±13.0 ms for T618I I_hERG_ (n = 5 and 6 cells respectively; p>0.5), indicating that activation time-course was similar for WT and T618I I_hERG_ during this protocol. In order to compare I_hERG_ deactivation time-course between WT and T618I hERG, I_tails_ elicited at −40 mV following voltage commands to +20 mV were fitted with a standard bi-exponential function. Figures 2Bi and Bii show respectively the mean fast and slow time-constants of deactivation (τ_f_ ‘fast tau’ and τ_s_ ‘slow tau’, respectively) for WT and T618I I_hERG_. Both fast and slow phases of deactivation were faster for T618I than WT I_hERG_ (evidenced by smaller tau values plotted in Figure 2B; p<0.05 and p<0.01 respectively for τ_f_ and τ_s_ versus WT). However, the relative proportion of fast and slow deactivation did not differ between WT and T618I I_hERG_ (quantified in Figure 2C as proportion of total deactivating current described by τ_f_).

### Effects of the T618I hERG Mutation on I_hERG_ Inactivation

**Figure 3:**
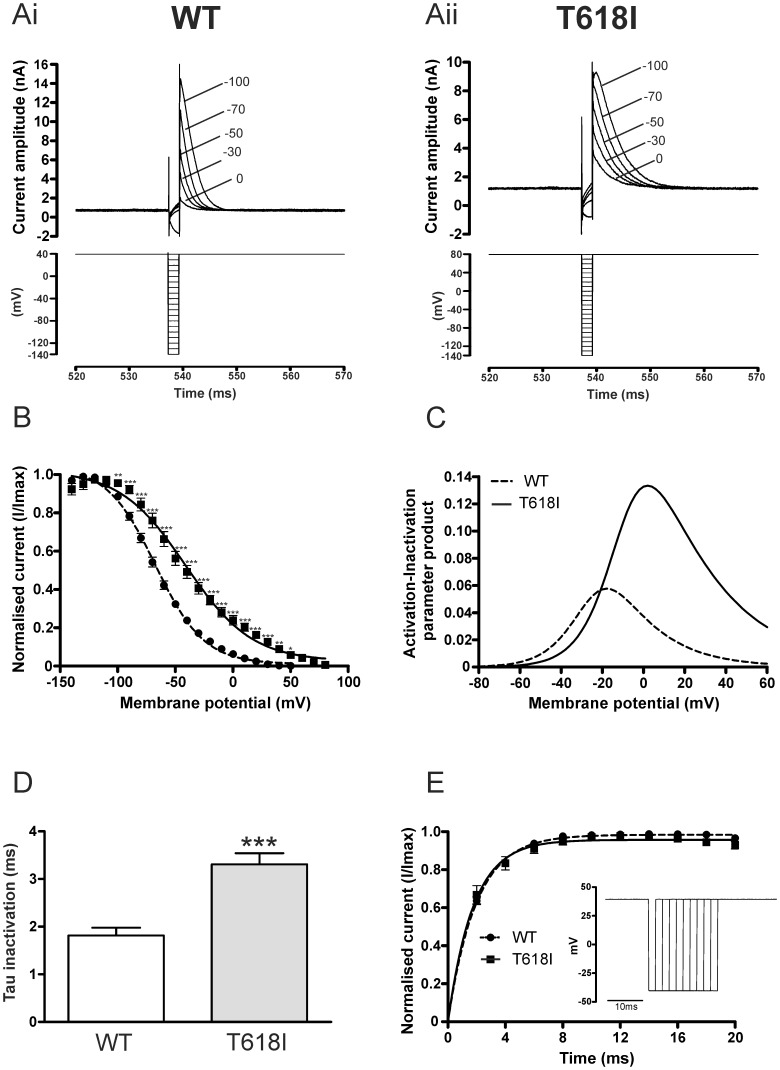
Voltage dependence of WT and T618I *I_hERG_* inactivation. (**Ai, Aii**) Upper traces show representative current records for WT I_hERG_ (Ai) and T618I I_hERG_ (Aii) elicited by voltage protocols shown in lower panels (see also ‘Results’ text). A more positive voltage (+80 mV) was used for the first and third steps for T618I hERG than for WT hERG (+40 mV) to ensure that complete inactivation occurred on membrane potential depolarisation. Note that for clarity of display only selected traces elicited by the protocol are shown. Numbers in Ai and Aii indicate command voltage at which currents recorded. (**B**) Plots against voltage (during the second step of the protocol) of I_hERG_ availability. Peak current values and availability plots were constructed as described previously [16], employing deactivation correction as per [31]. A Boltzmann equation fit to the data gave a V_0.5_ for inactivation of −67.2 mV (k = 21.0 mV) for WT and a V_0.5_ of −44.3 mV (k = 29.8) for T618I (n = 11 and 6 cells respectively). (**C**) ‘Window current’ for WT and T618I hERG. Derived activation and inactivation V_0.5_ and *k* values from Figures 1D and 3B were used with equations 1 and 2 to calculate activation and inactivation variables at 2 mV intervals between −80 and +60 mV. Window I_hERG_ was calculated as the activation-inactivation variable product at each voltage. (**D**) Bar charts comparing time constant of WT (n = 11) and T618I (n = 6) I_hERG_ inactivation, following brief hyperpolarisation to −140 mV. Inactivating currents were fitted with a standard mono-exponential function (**E**) Recovery from inactivation time-course for WT and T618I hERG (protocol shown as inset). The dashed gray line denotes mono-exponential fit to WT data (n = 7). The solid line denotes mono-exponential fit to T618I data, with the dotted line connecting these data at successive time points (n = 9). Asterisks in B and C denote statistical significance: *p<0.05; **p<0.01; ***p<0.001.

In order to characterize the effect of the T618I mutation on the voltage-dependence of I_hERG_ inactivation, voltage dependent availability of I_hERG_ was determined for WT- and T618I-hERG by applying voltage protocols used in prior investigations from our laboratory to study effects on inactivation of gain-of-function hERG mutations [16], [23]. These were comprised of an initial (500 ms) depolarizing step to activate and then fully inactivate I_hERG_, followed by brief (2 ms) repolarizing steps to a range of potentials to relieve inactivation to varying extents, followed by a third depolarization step that elicited a rapidly inactivating I_hERG_. The magnitude of peak current elicited by the third step reflected the extent of availability induced by the (second) repolarizing step. Similar to prior studies of gain-of-function hERG mutations performed in our laboratory [16], [23], in order to ensure complete inactivation of I_hERG_ during the initial step of the voltage protocol, for T618I I_hERG_ a depolarizing step to +80 mV was used, compared to +40 mV for WT I_hERG_. The lower panels of Figures 3Ai and Aii show the portion of the protocol that incorporated the repolarizing step and subsequent depolarization phases, with the upper panels showing corresponding I_hERG_ records. Peak current amplitudes were obtained by fitting the declining phase of the transient I_hERG_ records with a mono-exponential function and extrapolation to the beginning of the third pulse [16], [23]. The resulting values were normalized to the maximal current seen during the protocol and were plotted against repolarization step voltage. The availability/inactivation V_0.5_ value for WT I_hERG_ derived from a fit to the data with equation 2 was −65.5±2.2 mV with a *k* value of 19.8±0.6 (n = 11 cells). For T618I I_hERG_, the corresponding values were: V_0.5_ of −40.7±5.1 mV and *k* of 26.9±1.9 mV (n = 6 cells; and, respectively, p<0.01 and 0.001 versus control). For the sake of completeness, the data were further analysed by correction for deactivation using the method of Smith et al. [31] then plotted against voltage and fitted with equation 2 (Figure 3B), which gave V_0.5_ values of –67.2±2.0 and −44.3±5.1 mV respectively for WT and T618I I_hERG_ (p<0.01) and respective *k* values of 21.0±0.6 and 29.8±1.7 mV (P<0.001). Thus, I_hERG_ inactivation V_0.5_ was positively shifted by ∼+23 to +25 mV for T618I I_hERG_ compared to WT I_hERG_, with an accompanying 7 to 9 mV increase in *k* value. In order to establish the overall effects of altered steady-state voltage-dependent kinetics of the T618I mutation, we calculated ‘window current’ for WT and T618I hERG, as the activation-inactivation variable product across a range of voltages between −80 and +60 mV. Figure 3C shows that the I_hERG_ window was both positively shifted and significantly larger for T618I than WT I_hERG._

The time-course of development of inactivation for WT and T618I I_hERG_ was compared by mono-exponential fitting of the decline of transient currents elicited following the repolarizing step to −140 mV. This yielded τ-values for WT and T618I I_hERG_ inactivation of 1.86±0.17 ms and 3.32±0.28 ms respectively (Figure 3D; n = 11 and 6 respectively; p<0.01). To compare the rate of recovery from inactivation between the two channels, we used a protocol used in prior I_hERG_ studies ([23], [30] see also inset of Figure 3E): a 500-ms depolarisation to +40 mV was applied from a holding potential of −80 mV to activate and inactivate I_hERG_. Membrane potential was then repolarised to −40 mV for an increasing periods of time (between 2 and 20 ms) to induce recovery from inactivation. Transient currents were then subsequently elicited by a 100-ms depolarisation to +40 mV. Figure 3E shows plots of WT and T618I peak outward transient current magnitude against the duration of the repolarization step (with currents normalized to maximal current seen during the protocol). Fits to the data with a mono-exponential function gave τ values of 1.99±0.12 ms for WT (n = 7) and of 1.93±0.30 ms for T618I (n = 9) (p>0.05).

Collectively, the results from these experiments indicate that the T618I mutation induced a positive shift in the voltage-dependence of I_hERG_ inactivation, augmented the I_hERG_ ‘window’, slowed the time-course of development of I_hERG_ inactivation, but did not alter significantly the rate of recovery of I_hERG_ from inactivation.

### Effects of the T618I Mutation on I_hERG_ under Action Potential Voltage Clamp

**Figure 4:**
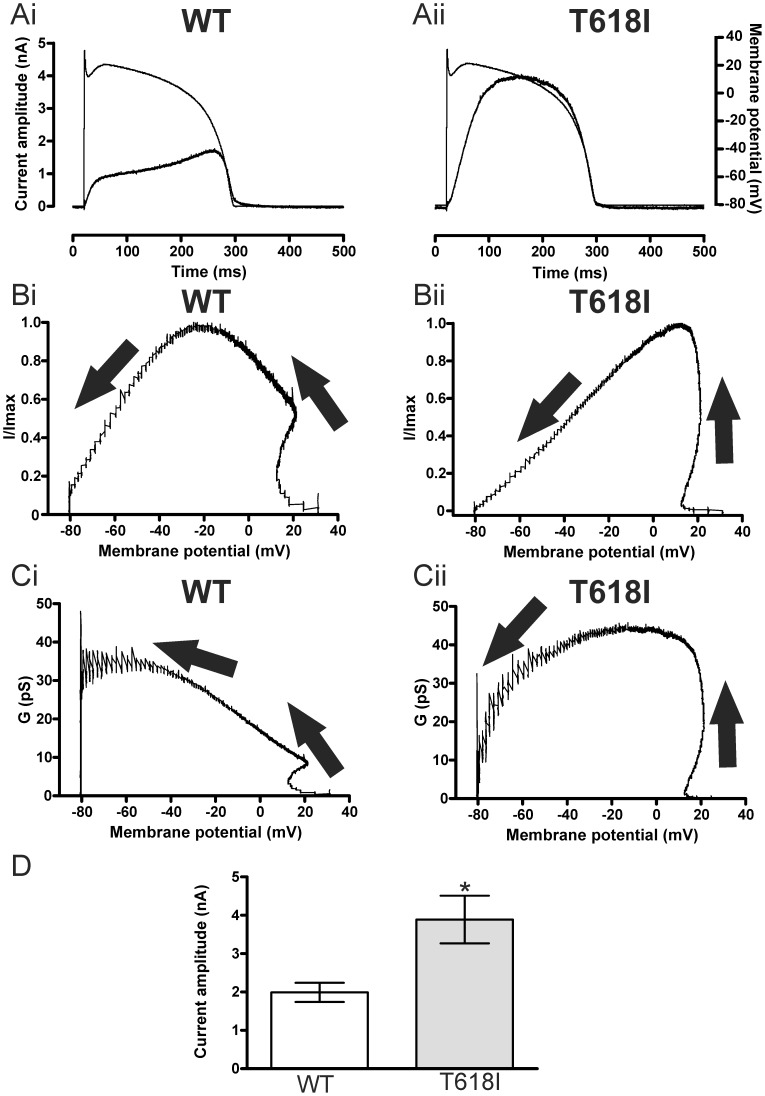
Action potential (AP) voltage clamp of WT and T618I I_hERG_. (**A**) I_hERG_ (after p/4 subtraction) elicited by ventricular AP command for WT (Ai) and T618I (Aii) I_hERG_. Currents are shown overlaid with the voltage protocol. (**B**) Instantaneous I–V relations for I_hERG_ elicited in A for WT (Bi) and T618I (Bii) I_hERG_. Current during the repolarising phase of the AP are plotted. Arrows denote direction of repolarisation. (**C**) Instantaneous conductance-voltage (G–V) relations for I_hERG_ elicited in A for WT (Ci) and T618I (Cii) I_hERG_ during AP repolarisation, Arrows denote direction of repolarisation. (**D**) Magnitude of peak repolarising current during AP voltage clamp, plotted for WT (n = 14) and T618I (n = 19) I_hERG_. * denotes statistically significant difference from WT at p<0.05.

Figure 4Ai shows a representative record of WT I_hERG_ elicited by a ventricular AP command (superimposed on the current trace in Figure 4Ai). As reported previously (e.g. [25], [30], [32]), the elicited current was comparatively small immediately on AP depolarisation, then increased progressively during the plateau phase of the AP, before declining during terminal repolarisation. Figure 4Aii shows similar recordings for T618I I_hERG_. The profile of current during the AP command differed from that for WT I_hERG_: current increased earlier during the AP command, peaking earlier during the AP plateau and then it declined during the latter part of the plateau phase. Figures 4Bi and Bii show representative normalized instantaneous current-voltage (*I–V*) relations for I_hERG_ during the repolarising phase of the AP command. Peak outward current was positively shifted by ∼ +35 mV for T618I I_hERG_ (from −30.7±1.2 mV for WT, to +5.1±2.1 mV for T618I hERG; *P*<0.001 versus WT). Example instantaneous conductance-voltage (*G-V*) relations (*cf* [16], [32], [33]) for WT- and T618I-hERG are shown in Figures 4Ci and Cii, respectively. As described previously [16], [32], [33], the macroscopic conductance of WT-hERG increased throughout the AP repolarisation phase, being maximal late in repolarisation (Figure 4Ci). In contrast, for T618I I_hERG_ conductance increased steeply early in repolarisation (between ∼+20 and −20 mV) and then progressively declined as the membrane potential followed the direction of membrane repolarisation. Figure 4D shows mean data for the maximal amplitudes of WT and T618I I_hERG_ during the applied ventricular AP command waveform, demonstrating a significantly greater (∼2-fold) maximal repolarising current when hERG channels incorporated the T618I hERG mutation. Considered collectively, the findings from our AP clamp experiments indicate that greater repolarising I_hERG_/I_Kr_ would be expected to occur earlier during ventricular APs in the setting of T618I-linked SQT1.

### Pharmacology of the T618I hERG Mutation

**Figure 5:**
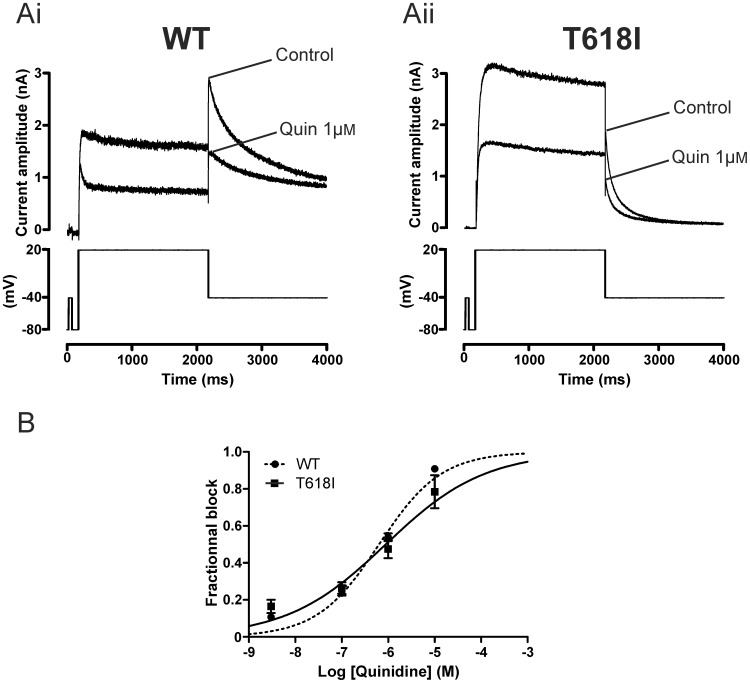
Effect of quinidine on WT and T618I I_hERG_ under conventional voltage clamp. (**A**) Upper traces show I_hERG_ elicited by voltage protocol shown in the lower panel (applied continuously once every 12 s) in control solution and after exposure to 1 µM quinidine (Quin). Ai shows data for WT I_hERG_, whilst Aii shows data for T618I I_hERG_. (**B**) Concentration response relations for inhibition of WT and T618I I_hERG_ by quinidine. Fractional inhibition of I_hERG_ was assessed for I_hERG_ tails at each of 4 concentrations (n = at least 5 cells per drug concentration). Steady-state effects were achieved within ∼2 minutes of drug application and measurements were made at ∼3 minutes.

Figure 5A compares the response of WT and T618I I_hERG_ to 1 µM of the Class Ia antiarrhythmic drug quinidine, using conventional voltage-clamp. Figures 5Ai and Aii show representative I_hERG_ traces elicited by the voltage protocol shown in the lower panels (a standard depolarising step protocol used in previous studies of I_hERG_ pharmacology from our laboratory (e.g. [23], [26], [34]). Tail current magnitude was measured relative to instantaneous current at −40 mV elicited by the brief (50 ms) depolarising step that preceded the voltage command to +20 mV in the absence and presence of the drug. 1 µM quinidine reduced WT I_hERG_ markedly, with 53.9±2.1% (n = 13) inhibition of the I_hERG_ tail evident (compatible with prior reports of a submicromolar IC_50_ under similar recording conditions [20], [35]). For T618I I_hERG_ the reduction in current was similar to that seen for the WT current, with 1 µM quinidine reducing T618I tail current magnitude by 47.6±5.0% (n = 8; NS versus WT). A range of quinidine concentrations between 10 nM and 10 µM were tested and concentration response relations constructed as shown in Figure 5B. The derived IC_50_ for WT I_hERG_ was 0.64 µM (for C.I. and n_H_ values see Table 1) whilst for T618I I_hERG_ the comparable value was 0.88 µM. Thus, the IC_50_ for I_hERG_ tail block by quinidine for T618I I_hERG_ was ∼ 1.4-fold that for WT I_hERG_ (see also Table 1).

**Table 1:** Pharmacology of the T618I hERG mutant studied with conventional voltage clamp.

Drug	WT I_hERG_ IC_50_ (µM)	WT I_hERG_ n_H_	T618I I_hERG_ IC_50_(µM)	T618I I_hERG_ n_H_	Fold IC_50_
**Quinidine**	0.64 (C.I 0.51–0.79)	0.65 (C.I 0.55–0.75)	0.88 (C.I 0.41–1.88)	0.41 (C.I 0.25–0.57)	1.4 (0.8–2.4)
**Disopyramide**	7.68 (C.I 6.32–9.34)	0.87 (C.I 0.66–1.07)	16.83 (C.I 8.56–33.09)	0.47 (C.I 0.30–0.64)	2.2 (1.4–3.5)
**D-Sotalol**	112.2 (C.I 91.7–137.3)	0.74 (C.I 0.57–0.91)	356.6 (C.I 305.6–416.1)	0.85 (C.I 0.70–1.00)	3.2 (3.0–3.3)
**Flecainide**	1.87 (C.I 1.56–2.25)	0.81 (C.I 0.68–0.94)	4.67 (C.I 3.06–7.13)	0.58 (C.I 0.45–0.71)	2.5 (2.0–3.2)

**Figure 6:**
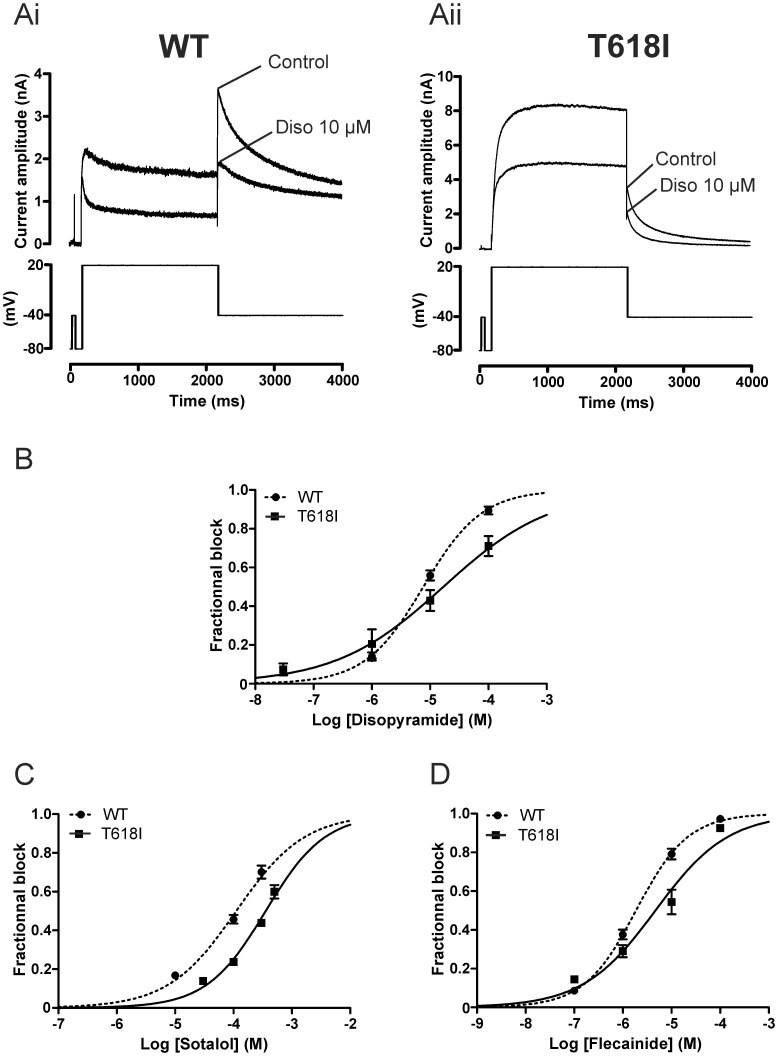
Effect of disopyramide, D-sotalol and flecainide on WT and T618I I_hERG_ under conventional voltage clamp. (**A**) Upper traces show I_hERG_ elicited by voltage protocol shown in the lower panel in control solution and after exposure to 10 µM disopyramide (Diso). Ai shows data for WT I_hERG_, whilst Aii shows data for T618I I_hERG_. Note different current scales in Ai and Aii. (**B**) Concentration response relations for inhibition of WT and T618I I_hERG_ by disopyramide. Fractional inhibition of I_hERG_ was assessed for I_hERG_ tails at each of 3 concentrations for WT I_hERG_ (n = 5 cells at 1 µM; 13 at 10 µM and 5 at 100 µM; incorporating data from [26], with additional data from a further 8 experiments for 10 µM disopyramide) and 4 for T618I I_hERG_ (n = 4 to 5 cells per concentration). (**C**) Concentration response relations for inhibition of WT and T618I I_hERG_ by D-sotalol. Fractional inhibition of I_hERG_ was assessed for I_hERG_ tails at each of 3 sotalol concentrations for WT I_hERG_ (n = 5 to 9 cells at each concentration) and 4 for T618I I_hERG_ (n = 5 to 6 cells per concentration). (**D**) Concentration response relations for inhibition of WT and T618I I_hERG_ by flecainide. Fractional inhibition of I_hERG_ was assessed for I_hERG_ tails at each of 4 flecainide concentrations for WT I_hERG_ (n = 5 to 12 cells per concentration) and T618I I_hERG_ (n = 5 to 6 cells per concentration).

IC_50_ and n_H_ values shown are derived from fits to concentration-response relations in Figures 5 and 6, obtained from fractional inhibition of I_hERG_ using a voltage step protocol (shown in Figures 5A and 6A). Columns show mean values and 95% confidence intervals (C.I). The right-hand column expresses the ratio of the T618I IC_50_ to the WT IC_50_ value, to one decimal place. The numbers in parentheses in the right hand column represent the range of ratio values for the ± C.Is for derived T618/WT IC_50_s.

The Class Ia antiarrhythmic drug disopyramide has been found to be effective against the N588K I_hERG_ SQT1 mutation [20], [21], but its effects on T618I hERG are unknown. Therefore we tested the effects of disopyramide on the T618I hERG mutant (Figure 6). Figures 6Ai and Aii show representative I_hERG_ traces in the absence and presence of the drug, with the protocol shown in lower panels. As expected from previous studies [20], [26], [36], 10 µM disopyramide reduced WT I_hERG_ by 55.9±2.6% (n = 13) whereas it reduced T618I I_hERG_ by 42.9±5.4% (n = 5,p<0.05 versus WT). Three other disopyramide concentrations were tested on T618I mutant channels and concentration response relations constructed as shown in Figure 6B. For T618I I_hERG_ the disopyramide IC_50_ was 16.83 µM whilst for WT I_hERG_ inhibition the corresponding value was 7.68 µM. Thus, the IC_50_ for T618 I_hERG_ tail inhibition by disopyramide was ∼2.2 fold that for the WT channel.

Similar experiments were also conducted with the Class III antiarrhythmic drug D-sotalol and the Class Ic antiarrhythmic drug flecainide. Figures 6C and 6D show the concentration-response relations for inhibition of WT and T618I I_hERG_ by these drugs. For D-sotalol (Figure 6C) the derived IC_50_ for WT I_hERG_ was 112.2 µM whilst for T618I I_hERG_ the comparable value was 356.6 µM (∼3.2-fold that for WT I_hERG_). For flecainide, the derived IC_50_ for WT I_hERG_ was 1.87 µM whilst that for T618I I_hERG_ was 4.67 µM (∼ 2.5-fold that for WT I_hERG_). Results obtained under conventional voltage clamp for all four drugs are summarized in Table 1.

**Figure 7:**
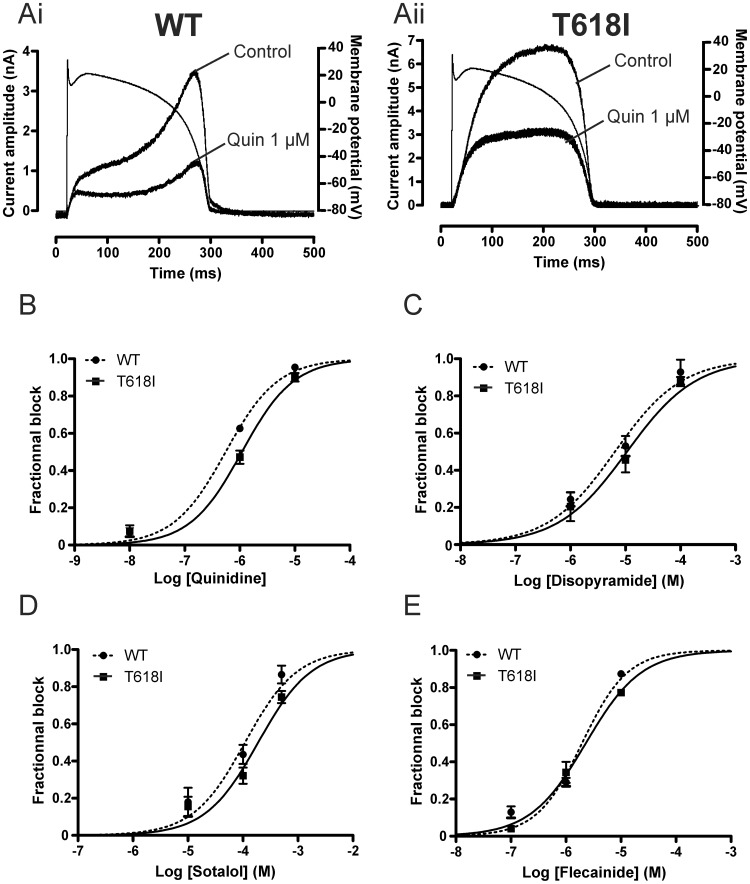
Pharmacology of WT and T618 I_hERG_ under AP voltage clamp. (**A**) Shows representative traces (after p/4 subtraction) of WT (Ai) and T618I (Aii) I_hERG_ elicited by AP voltage clamp protocol (overlain) in control solution and after exposure to 1 µM quinidine. Note different current scales in Ai and Aii. AP commands were applied at 1 Hz. (**B–D**) Concentration response relations for inhibition of WT and T618I peak repolarising current observed during AP clamp by quinidine (B; 3 concentrations tested, n = 4 to 5 cells per concentration); disopyramide (C; 3 concentrations tested, n = 4 to 5 cells per concentration); D-sotalol (D; 3 concentrations tested, n = 4 to 5 cells per concentration); flecainide (E; 3 concentrations tested, n = 4 to 5 cells per concentration).

The limited data currently available on T618I hERG pharmacology appear to suggest some difference in the effect of the mutation on inhibition of pulse and tail currents by 1 µM quinidine and 500 µM sotalol, during conventional voltage clamp [24]. Ventricular APs involve dynamic changes in membrane potential that influence the profile of observed current; therefore we conducted additional experiments in which concentration-response relations for the 4 drugs examined under conventional voltage clamp were also determined from ventricular AP clamp experiments. For these, the percentage of inhibition of peak I_hERG_ during the AP waveform for three different drug concentrations was calculated for each drug. Concentration-response relations were then constructed as shown in Figure 7. Figures 7Ai and Aii show representative current traces for WT and T618I I_hERG_ in the presence and absence of quinidine; the ventricular AP command is superimposed over each set of traces. In this example, 1 µM quinidine reduced maximal I_hERG_ during repolarisation by 67% for WT I_hERG_ and 56% for T618I I_hERG_. Figures 7B–E show concentration-response curves for inhibition of maximal I_hERG_ during repolarisation by quinidine, disopyramide, sotalol and flecainide (Figures 7B–E respectively), whilst Table 2 summarises numerical data for IC_50_ and n_H_ values. The derived IC_50_ values for WT and T618I I_hERG_ inhibition by quinidine (Figures 7A,B) were, respectively, 0.55 µM and 1.09 µM (∼2.0 fold the WT value). For disopyramide (Figure 7C), the WT I_hERG_ IC_50_ was 6.47 µM and that for T618I I_hERG_ was 10.65 µM (1.6-fold the WT value). For D-sotalol (Figure 7D) the IC_50_ for WT I_hERG_ was 109.5 µM, whilst that for T618I I_hERG_ was 189.2 µM (∼1.7-fold that for WT I_hERG_). Finally, we found flecainide (Figure 7E) to inhibit WT and T618I I_hERG_ with an IC_50_ of 1.96 µM and of 2.29 µM (1.2 fold the WT value) respectively. Thus, under AP clamp all four drugs exhibited comparatively modest attenuation of their inhibitory action with the T618I mutation.

**Table 2:** Pharmacology of the T618I hERG mutant studied with action potential voltage clamp.

Drug	WT I_hERG_ IC_50_ (µM)	WT I_hERG_ n_H_	T618I I_hERG_ IC_50_(µM)	T618I I_hERG_ n_H_	Fold IC_50_
**Quinidine**	0.55 (C.I 0.43–0.71)	0.91 (C.I 0.65–1.18)	1.09 (C.I 0.82–1.46)	0.92 (C.I 0.59–1.26)	2.0 (1.9–2.1)
**Disopyramide**	6.47 (C.I 3.76–11.12)	0.71 (C.I 0.40–0.99)	10.65 (C.I 5.73–19.80)	0.69 (C.I 0.35–1.05)	1.6 (1.5–1.8)
**D-Sotalol**	109.5 (C.I 70.7–169.6)	0.92 (C.I 0.47–1.37)	189.2 (C.I 133.3–268.6)	0.90 (C.I 0.56–1.25)	1.7 (1.6–1.9)
**Flecainide**	1.96 (C.I 1.49–2.59)	1.05 (C.I 0.79–1.30)	2.29 (C.I 1.72–3.05)	0.86 (C.I 0.67–1.05)	1.2 (1.1–1.2)

IC_50_ and n_H_ values shown are derived from fits to concentration-response relations in Figure 7, obtained from fractional inhibition of I_hERG_ using a voltage step protocol (shown in Figure 7A). Columns show mean values and 95% confidence intervals (C.I). The right-hand column expresses the ratio of the T618I IC_50_ to the WT IC_50_ value, to one decimal place. The numbers in parentheses in the right hand column represent the range of ratio values for the ± C.Is for derived T618/WT IC_50_s.

## Discussion

To our knowledge, the present study is the first to have established the effects of the T618I hERG mutation on the profile of I_hERG_ during physiological waveforms. Although limited (single concentration) *in vitro* data have been reported for racemic sotalol and quinidine [24], the present study is the first to provide concentration response data for any drug against T618I hERG and it is also the first to provide any *in vitro* data for T618I I_hERG_ inhibition by disopyramide, D-sotalol and flecainide.

### Effects of the T618I Mutation on I_hERG_

Although the recent study by Sun and colleagues is the first report of the occurrence of the T618I hERG mutation in a clinical context [24], one other investigation has utilised this mutation in the study of the role of a nearby S5 residue (H562) that is able to interact with the pore helix [37]. In that study, T618I I_hERG_ was shown to exhibit both increased currents at positive voltages and reduced tail currents compared to pulse currents following positive voltage commands [37]. These features are in accord with the subsequent report by Sun and colleagues and with our own data. However, Lees-Miller and colleagues reported a significant (>+30 mV) positive shift in I_hERG_ activation V_0.5_ for T618I hERG [37], whilst Sun *et al.* reported a small (∼−5 mV) negative shift in activation V_0.5_ compared to WT I_hERG_ [24]. Both studies were conducted using HEK cells for hERG channel expression, whilst Lees Miller *et al.* performed measurements at 36°C [37] and the recording temperature for the study by Sun *et al.* was not given [24]. Thus, the reason for the apparently opposite observations in the two studies in respect of activation V_0.5_ is not clear. In our experiments, using a similar expression system and recording at 37°C, there was a +15 mV shift in activation V_0.5_, which is in good qualitative agreement with the findings of Lees Miller *et al.* [37], but differs from the negative activation shift of V_0.5_ reported by Sun and colleagues [24]. However, unlike Lees-Miller *et al.* [37] and in accord with Sun and colleagues [24], we saw a significant effect of the mutation on I_hERG_ deactivation time-course, with both τ_f_ and τ_s_ time constants of T618I I_hERG_ deactivation smaller than those for WT I_hERG_. Also in accord with Sun and colleagues [24], we did not observe any significant alteration to the rate of I_hERG_ activation for the T618I hERG mutant. The shift in voltage dependence of I_hERG_ inactivation (availability) seen here (Figures 3A,B) is in qualitative agreement with positive shifted inactivation reported by Sun *et al.* [24], as is the positively shifted region of negative slope in the end pulse I-V relation (Figure 1C). However, the extent of positive shift in inactivation V_0.5_ (∼+23–25 mV) seen here was smaller than that reported by Sun and colleagues (∼+50 mV); the reason for this difference is at present not clear. Nevertheless. the shift steady-state inactivation seen here is demonstrably sufficient to lead to a significant functional impact: ‘window current’ calculations based on our derived activation and inactivation V_0.5_ and *k* values revealed significant augmentation, as well as positively shifted peak, of the I_hERG_ window for T618I hERG when compared with WT hERG (Figure 3C). Slowing of time-dependent development of inactivation of I_hERG_ (Figure 3D) may have a synergistic effect in permitting greater I_hERG_ to flow. A modulatory effect of the T618I mutation on I_hERG_ inactivation is not entirely unexpected, given that mutation of the nearby S620 residue (to S620T) has been established to abolish hERG channel inactivation (e.g. [38], [39]), although in contrast to the S620T mutation it is clear that the T618I mutation produces a more modest, partial attenuation of I_hERG_ inactivation.

### Pharmacology of T618I hERG

It is well established that the inactivation process is important for binding to the hERG channel of a range of drugs, but it is also the case that not all drugs are equally dependent upon inactivation for binding to the channel to occur (e.g. [18], [20], [21], [38]–[42]). The SQT1 N588K mutation has been shown to lead to markedly elevated IC_50_ values for I_hERG_ blockade by methanesulphonanilide compounds including sotalol (20-fold WT I_hERG_ IC_50_ for D-sotalol) and E-4031 (∼11.5-fold WT I_hERG_ IC_50_), whilst those for quinidine (∼3.5–6-fold WT I_hERG_ IC_50_) and disopyramide (1.5-fold WT I_hERG_ IC_50_) are comparatively little affected [18], [20]. In the present study, the T618I hERG mutation elevated the IC_50_ for D-sotalol by ∼3 fold under conventional voltage clamp, which is substantially less than that seen for N588K hERG [18]. With a voltage protocol similar to that used in the present study, I_hERG_ N588K hERG availability was found to be positively shifted by ∼+62 mV [16]. In the present study, the shift in the voltage dependence of T618I I_hERG_ inactivation was +∼23-+25 mV. It therefore seems likely that the smaller effect of the T618I mutation in attenuating I_hERG_ block by D-sotalol can be attributed to the ability of the T618I channel to inactivate to a greater extent than has been found to be the case for N588K hERG. In this regard, it is noteworthy that the results of experiments in which different inactivation mutations have been combined in order to titrate hERG inactivation suggest that I_hERG_ blocking potency is not related to inactivation in a linear fashion. Thus, for both quinidine and disopyramide single mutations that reduced macroscopic I_hERG_ inactivation to ∼20% produced only modest (1.5 and 3.5 fold) reductions in the potency of disopyramide and quinidine, whilst double-mutations that reduced inactivation to <10% led to elevations of IC_50_ by 6.5 and 7-fold respectively [21]. For T618I hERG in this study, the IC_50_ for quinidine under conventional voltage clamp was ∼ 1.4 that of the WT channel, whilst for disopyramide it was ∼2.2-fold that of the WT channel. The attenuation of I_hERG_ inactivation by the T618I mutation therefore appears to be insufficient to interfere dramatically with drug binding. At the same time, the greater effect of this mutation on disopyramide’s potency under conventional voltage clamp than that seen previously for the N588K mutation (which impairs inactivation to a greater extent than does T618I [15], [16], [21], [24]) suggests that other effects of the mutation on channel conformation as well as upon inactivation *per se* may contribute to its overall effect on disopyramide binding. To our knowledge there are no prior data available on effects of inactivation-attenuating hERG mutants on I_hERG_ blocking potency of flecainide. A prior study from our laboratory has shown that the characteristics of flecainide inhibition of WT I_hERG_ are qualitatively similar to those of the Class Ia antiarrhythmic quinidine and of another Class Ic drug, propafenone [35]. Given that quinidine, disopyramide and propafenone have all been shown to exhibit comparatively little dependence on hERG channel inactivation to exert their inhibitory effects [20], [21], [36], [42] and also that the T618I mutation produced only a modest effect on I_hERG_ blocking potency in this study, it seems reasonable to conclude that hERG channel inactivation is not a major determinant of flecainide potency against hERG. Further experiments on attenuated inactivation mutants are required to determine unequivocally whether or not this is the case.

We also compared between WT and T618I hERG the potency of I_hERG_ inhibition under AP clamp, for each of the drugs studied under conventional voltage clamp. It is known that drug inhibitory potency against I_hERG_ can vary depending on stimulus protocol [34], [43], [44]. In our experiments, both stimulus waveform (step versus AP command) and stimulus frequency (repetitive pulsing once every 12 s versus once every second -to apply APs at a physiological rate) differed between the protocols used to obtain the data in Figures 6 and 7. However, the IC_50_ values for WT I_hERG_ inhibition by any of the drugs studied did not differ greatly between conventional and AP clamp protocols (see Tables 1 and 2). In general, however, differences between IC_50_ values obtained with conventional and AP clamp protocols were greater for T618I hERG, although the C.I range for IC_50_s with the two protocols showed either some overlap (quinidine, disopyramide, D-sotalol) or little separation (flecainide). The range of T618I/WT IC_50_ ratio values was found to be somewhat smaller (1.2–2.0) under AP clamp than under conventional voltage clamp (1.4–3.2), with a marked reduction in this for D-sotalol. Contributory factors to this may be intrinsic voltage-dependence of inhibition [34]–[36] together with the occurrence (and hence measurement) of peak I_hERG_ at a comparatively positive voltage for T618I compared to WT I_hERG_ during the AP waveform (and compared to the measurement voltage (−40 mV) for T618I I_hERG_ tails under conventional voltage clamp), and a greater sensitivity of drug block to duty-cycle (rate) for the mutant. On the basis of our findings, future detailed investigation of effects of T618I hERG kinetics on channel block are likely to be instructive in this regard, though are beyond the intended scope of the present study.

One puzzling aspect of our pharmacology data is that for the Class I drugs studied, n_H_ values derived from concentration-response relations obtained under conventional voltage clamp were substantially lower (<0.5 for quinidine and disopyramide) for T618I than for WT I_hERG_, whilst this was not the case under AP clamp (compare Tables 1 and 2). The low n_H_ values under conventional voltage clamp do not appear to be attributable to voltage-drop down uncompensated series resistance for T618I I_hERG_ recordings: estimated voltage drop was lower for quinidine (2.31±0.37 mV; n = 16) than for D-sotalol (7.61±0.37 mV; n = 21), although the n_H_ value was higher for D-sotalol than for quinidine (Table 1). On the other hand, were the marked reduction in n_H_ for quinidine and disopyramide strongly reflective of altered drug-channel interaction due to the T618I mutation it might be anticipated also to occur for data from AP clamp experiments and this was not the case. The basis for the apparently low n_H_ for quinidine and disopyramide for T618I I_hERG_ remains unexplained at the present time. Arguably, the more (patho)physiologically relevant pharmacological data are those obtained under AP clamp at a physiologically relevant rate; the major conclusion from those data (Figure 6 and Table 2) is that under these conditions the T618I mutation did not produce a large attenuation of inhibitory potency for any drug studied.

### Clinical Relevance

In this study we observed that, under AP clamp, T618I mutant I_hERG_ exhibited an altered current profile, peaking earlier during the AP plateau than was the case for WT I_hERG_. Previous studies in which the SQT1 N588K hERG mutant has been studied under ventricular AP clamp have shown an inverted U or bow-shaped current profile peaking at ∼+20 mV, consistent with the occurrence of little inactivation over physiologically relevant membrane potentials [13], [15], [16], [33]. The N588K hERG mutation produces a greater attenuation of I_hERG_ inactivation than does T618I hERG and our AP clamp data are suggestive of an electrophysiological phenotype for T618I hERG during the ventricular AP that is intermediate between those of WT and N588K I_hERG_. Accordingly, the effect of the T618I mutation in accelerating ventricular AP repolarisation can also be predicted to be less than that of N588K hERG. This is in agreement with the less extensive QT_c_ interval shortening for SQT1 patients with the T618I mutation (mean in affected individuals of 316 ms) [24] than those with the N588K mutation (QT_c_ of ≤ 300 ms in the first two SQT1 genotyped families [13] and a QT of 230 ms in the proband of a third family [14]). The normally slow deactivation of I_Kr_/I_hERG_ can contribute to resting membrane conductance and protection from premature depolarisation immediately after completion of ventricular AP repolarisation; in pathological settings accelerated I_Kr_ deactivation may increase excitability early in diastole [30], [45]. Whether or not the faster deactivation of T618I than WT hERG is able to contribute abbreviated refractory period and susceptibility to programmed simulation (as clinically observed for SQT1 patients with the T618I mutation [24]) remains to be established, but warrants future *in silico* investigation [30], [46].

A first line treatment for the SQTS is the use of implantable defibrillators (ICDs) to protect against sudden death, although ICD use itself carries the risk of inappropriate shocks [47]. A recent report of long term follow up of SQTS patients noted that 58% of patients with ICDs had device-related complications [48]. The same report [48] notes that the T618I SQT1 mutation has now been found in a second family in addition to that originally identified by Sun and colleagues [24]. Pharmacological therapy is therefore attractive both for patients in whom ICDs are not fitted and as an adjunct therapy to reduce arrhythmic events and restore QT intervals towards normal. Our findings extend those previously obtained at a single (>70% blocking) quinidine concentration [24]; collectively the full concentration response data obtained with both conventional voltage and AP clamp protocols indicate that quinidine largely retains its potency against T618I I_hERG_. The available *in vitro* data therefore indicate that quinidine is likely to be beneficial in T618I-linked SQT1. Concordant with this, hydroquinidine has recently been reported to have a positive effect on QT_c_ intervals in T618I hERG carriers [48]. However, whilst the available evidence from long-term follow up of SQTS patients is that (hydro)quinidine is also effective in arrhythmia prophylaxis [48], diminishing availability of quinidine [49] makes it attractive to find alternative pharmacological therapies for use in SQTS patients. Disopyramide is effective against the N588K mutation *in vitro* [20] and has shown benefits in SQT1 patients [19]. Our experiments indicate that although there is a modest reduction in disopyramide potency for T618I I_hERG_ both during conventional and AP voltage protocols, our data indicate that some I_hERG_/I_Kr_ blockade can nevertheless be expected to occur within the clinical concentration range (∼6–8 µM; [50]). Thus, disopyramide may be worthy of investigation as a potential treatment for T618I-linked SQT1. Sun *et al.* have suggested that T618I hERG carriers may be less resistant to drugs like sotalol than had been previously found for N588K-linked SQT1 [24]. Our data with D-sotalol, particularly those obtained under AP voltage clamp, support this proposition; the reduction in I_hERG_ blocking potency of D-sotalol by T618I hERG appears to be substantially less than that produced by N588K hERG [18]. It is possible, therefore, that sotalol may be worthy of clinical investigation for T618I-linked SQT1. Future *in vitro* work may also be warranted to determine whether higher affinity methanesulphonanilide Class III drugs than sotalol that are in clinical use (ibutilide, dofetilide) are able to exert some inhibition of T618I at clinically relevant concentrations. Of particular note, flecainide was found to exert marked inhibition of both WT and T618I I_hERG_ at concentrations relevant to clinical serum levels (0.5 to 2.4 µM; [51]), with little difference between WT and T618I I_hERG_ IC_50_ under AP clamp. Flecainide has been tested previously in a group of SQT1 (N588K hERG) patients unresponsive to sotalol but responsive to hydroquinidine [52]. In that study it was found to produce a small prolongation of QT interval in some patients, which was largely attributable to QRS interval lengthening [52]. To our knowledge, comparable data are lacking in patients with the T618I hERG SQT1 mutation. However, on the basis of our findings flecainide may warrant investigation in this group. Sun and colleagues have also provided evidence that a >70% blocking concentration of quinidine reduces the inactivation shift for T618I I_hERG_ [24]. However, in our AP clamp experiments none of the drugs studied produced any consistent correction of T618I I_hERG_ profile during a physiological waveform; this suggests that potential benefits of the drugs studied here for QT intervals in patients with the T618I hERG mutation are likely to be attributable to reduction in total repolarising I_hERG_/I_Kr_, without restoration of the current’s normal time- and voltage- dependent profile during ventricular APs.
